# Summarizing Online Patient Conversations Using Generative Language Models: Experimental and Comparative Study

**DOI:** 10.2196/62909

**Published:** 2025-04-14

**Authors:** Rakhi Asokkumar Subjagouri Nair, Matthias Hartung, Philipp Heinisch, Janik Jaskolski, Cornelius Starke-Knäusel, Susana Veríssimo, David Maria Schmidt, Philipp Cimiano

**Affiliations:** 1 Cognitive Interaction Technology Center Faculty of Technology Bielefeld University Bielefeld Germany; 2 Semalytix GmbH Bielefeld Germany

**Keywords:** patient experience, online communities, summarizing, large language models

## Abstract

**Background:**

Social media is acknowledged by regulatory bodies (eg, the Food and Drug Administration) as an important source of patient experience data to learn about patients’ unmet needs, priorities, and preferences. However, current methods rely either on manual analysis and do not scale, or on automatic processing, yielding mainly quantitative insights. Methods that can automatically summarize texts and yield qualitative insights at scale are missing.

**Objective:**

The objective of this study was to evaluate to what extent state-of-the-art large language models can appropriately summarize posts shared by patients in web-based forums and health communities. Specifically, the goal was to compare the performance of different language models and prompting strategies on the task of summarizing documents reflecting the experiences of individual patients.

**Methods:**

In our experimental and comparative study, we applied 3 different language models (Flan-T5, Generative Pretrained Transformer [GPT], GPT-3, and GPT-3.5) in combination with various prompting strategies to the task of summarizing posts from patients in online communities. The generated summaries were evaluated with respect to 124 manually created summaries as a ground-truth reference. As evaluation metrics, we used 2 standard metrics from the field of text generation, namely, Recall-Oriented Understudy for Gisting Evaluation (ROUGE) and BERTScore, to compare the automatically generated summaries to the manually created reference summaries.

**Results:**

Among the zero-shot prompting–based large language models investigated, GPT-3.5 performed better than the other models with respect to the ROUGE metrics, as well as with respect to BERTScore. While zero-shot prompting seems to be a good prompting strategy, overall GPT-3.5 in combination with directional stimulus prompting in a 3-shot setting had the best results with respect to the aforementioned metrics. A manual investigation of the summarization of the best-performing method showed that the generated summaries were accurate and plausible compared to the manual summaries.

**Conclusions:**

Taken together, our results suggest that state-of-the-art pretrained language models are a valuable tool to provide qualitative insights about the patient experience to better understand unmet needs, patient priorities, and how a disease impacts daily functioning and quality of life to inform processes aimed at improving health care delivery and ensure that drug development focuses more on the actual priorities and unmet needs of patients. The key limitations of our work are the small data sample as well as the fact that the manual summaries were created by 1 annotator only. Furthermore, the results hold only for the examined models and prompting strategies, potentially not generalizing to other models and strategies.

## Introduction

### Background

For many patients, the World Wide Web plays an important supporting role in coping with their condition. In fact, social media sites, digital support groups, and forums as well as social networks play an important role in the life of patients as sharing and consuming experiences with peers provides emotional, informational, and psychological support [1]. It has been indeed shown that sharing health-related experiences in online communities has an effect of empowerment on patients, increasing their subjective and psychological well-being, leading to increased self-management and control [1].

In addition to being used for receiving support, social media is increasingly used by patients to educate themselves on a disease and to find the hospitals or physicians most capable of treating their condition [2]. When engaging with social media, users are willing to disclose very intimate information [3]. In fact, it has been shown that the anonymity of many social media sites increases the level of self-disclosure [4]. The consumer survey by the PwC Health Research Institute [5] has indeed shown that >30% of respondents would be comfortable having their social media conversations monitored if those data could help identify ways to improve their health. In a study with adults presenting to an academic, urban emergency department, Padrez et al [6] found that, among patients with a social media profile, 71% consented to sharing their social media data to compare it to their electronic medical records. These figures convey that a substantial proportion of patients are willing to share their social media data for research purposes if the data contribute to improving their condition and those of their peers. A recent study confirmed these previous results, showing that participants would be willing to donate some of their digital data to researchers and clinicians in pursuit of health-related insights [7].

Against this background—the World Wide Web and social media playing an empowering role in patients’ lives and, at the same time, patients being willing to donate their data if this contributes to improving their and others’ health condition—an important question is how relevant insights can be obtained from the data. The Food and Drug Administration has identified social media data as one important source of “patient experience data,” that is, data that can foster understanding of the unmet needs and priorities of patients as a basis to design more effective clinical studies; support Outcomes Research activities; and, ultimately, develop better drugs that actually improve patients’ lives, well-being, and functioning [8]. In fact, methodologies for patient listening are quite mature and are widely used in patient-focused drug development to automatically analyze social media content as a basis to understand which symptoms are most burdensome to patients; how they experience existing treatments, which outcomes matter to them, and, overall, how their quality of life is affected by the disease [9].

However, these methodologies mostly provide quantitative insights as they rely on the automatic analysis of social media content using techniques from natural language processing (NLP), extracting key information and summarizing it statistically to obtain an understanding of trends at the level of an entire patient population or patient cohort of interest. A frequent problem is how to obtain a more qualitative understanding of how the disease affects patients’ daily functioning and living. Most NLP methods, in fact, can extract single entities or concepts from textual data, but there is a lack of methods to analyze textual data at a more qualitative level in terms of the key messages that can be derived from a collection of texts. Summarization techniques, especially from abstractive summarization, can be used for this purpose, but so far research on how well posts from patients in online communities can be faithfully summarized is missing. While it is possible to manually analyze sample posts, there is the risk of missing relevant aspects, and the task of summarizing all the content manually is infeasible. Thus, there is a need for more scalable methods to derive qualitative insights from online patient experience data.

Considering recent developments in the field of large language models (LLMs), in this paper, we ask the question of to what extent existing pretrained LLMs can be used to aid the analysis and summarization of patient-authored social media content. LLMs are machine-learned models that have been “pre-trained” on large amounts of text [10] and later either fine-tuned for specific tasks [10-12] or trained to determine a suitable response to a certain instruction. The latter has been referred to as “alignment” and is typically done using reinforcement learning [13]. Overall, LLMs have been shown to perform very well on tasks such as summarization [14,15], sentiment analysis [16], question answering [17], and machine translation [12]. While excelling at many tasks, LLMs have also been shown to lack robustness and react too sensitively to small perturbations in the input and to be misled by so-called “adversarial examples” [18]. Furthermore, they have been shown to extrapolate too creatively in some cases, generating output that is factually wrong. This phenomenon has been sometimes referred to as “hallucination” [19].

### Objectives

On the basis of the need for advanced methods to summarize the patient experience to yield qualitative insights and the promise of LLMs for many tasks, in this paper, we explicitly address the following question: can state-of-the-art LLMs be used to accurately summarize the experience of patients as shared in online data sources?

For this purpose, we experimentally compared different pre-trained LLMs: 2 from the Generative Pretrained Transformer (GPT) family (GPT-3 and GPT-3.5 [OpenAI]) and Flan-T5 (Google). While our focus was on using pretrained language models (LMs) that are prompted via instructions, we also carried out fine-tuning experiments to examine the impact of fine-tuning. We experimentally investigated several prompting strategies—zero-shot, 1-shot, and 3-shot learning—both alone and in combination with directional stimulus prompting (DSP) and chain of thought (CoT) prompting.

For this study, we focused on breast cancer and collected self-reported patient experience data from 5 forums, grouping the documents according to mentions of key symptoms and treatments. The data collected comprise user posts from 5 leading web forums in which patients with breast cancer discuss their experiences with peers. The 5 sources were manually identified in a search process with the aim to identify the most relevant breast cancer forums.

The texts generated by the LLMs were evaluated with respect to a dataset of 127 manually produced reference summaries that were provided by one of the authors with years of expertise in analyzing patient experience data from social media. The generated texts were evaluated in terms of standard text generation metrics, including the Recall-Oriented Understudy for Gisting Evaluation (ROUGE) metrics and BERTScore. These metrics essentially compute the overlap between the automatically generated texts and the manually created references and, thus, provide an estimate of the quality of the summaries. In a case study, we discuss the output of different LLMs to obtain a sense of how these models are actually able to summarize the conversations.

Our results show that advanced prompting techniques substantially improve the ability of generative LMs to summarize online patient comments efficiently.

## Methods

### Overview

The research design corresponds to an experimental and comparative study. It corresponds to an experimental study in that it involved experiments in which the ability of LLMs to adequately summarize patient posts was experimentally evaluated by comparing automatically generated summaries to manually created summaries in a series of experiments. This study was also comparative as it evaluated the performance of different LLMs and prompting strategies in comparison to each other. The statistical validity of the results was assessed by computing their statistical significance using a 2-tailed *t* test and reporting *P* values comparing the significance of the results of the best-performing approach to that of the results of the other approaches. While, in most settings, we relied on off-the-shelf pretrained LLMs, we also tested the effect of fine-tuning on the task.

### Dataset and Task Description

For this experimental and comparative study, data were crawled from different web-based forums in which patients with breast cancer discussed their disease experience with peers. The data were collected from the following public forums based on a search by the authors for the most prominent forums related to breast cancer available on the web: breastcancer.org, HealthUnlocked, her2support.org, and Macmillan Cancer Support. Data were automatically obtained via Socialgist [20] as a data provider and automatically analyzed to identify posts of individuals who had self-disclosed being patients with breast cancer using the methodology described previously by Spies et al [21]. The processing consisted of anonymization; entity extraction and linking via a knowledge graph; detection of symptoms, treatments, and quality of life mentions; detection of the severity of the symptoms; and detection of the impact of symptoms on the quality of life of the patients. In addition, demographic information involving age and gender was extracted if explicitly mentioned in the posts.

This yielded a dataset comprising 145,460 documents from 1146 patients. The preprocessing revealed the most frequent symptoms (n=8) and most frequent treatments used (n=119). They are listed in Multimedia Appendix 1. The data originated from a time frame between August 2016 and July 2022. As expected from a population with breast cancer, 99.5% (1089/1146) of the patients were female (male individuals can develop breast cancer in rare cases).

Before analysis, the data were automatically anonymized to remove mentions of person names, emails, URLs, telephone numbers, and addresses. A sample analysis showed that the accuracy of anonymization was 99.5%. For this purpose, we relied on the anonymization solution provided by Private AI [22].

The summarization corpus underlying this study was constructed as follows. For each topic in the dataset (where “topic” refers to one of the key symptoms of breast cancer or one of the existing breast cancer treatments as listed in Multimedia Appendix 1), all documents containing at least one mention of the topic were collapsed into a topic-specific subcorpus. If multiple topics were mentioned within a document, it became part of several subcorpora accordingly. This resulted in 127 topic-specific subcorpora derived from 8 key symptoms and 119 treatments. The number of documents per topic-specific subcorpus varied between 1 and 44, with a median of 12.

The summarization task can be seen as an instance of multidocument summarization as it consists of generating a concise summary for each of the 127 topic-specific subcorpora in the summarization corpus. The main challenges for multidocument summarization are, in addition to the fact that the input can be very long, the fact that the content of the documents to be summarized might be overlapping, complementary, or even inconsistent in the worst case [23].

As a gold standard for reference, 1 coauthor of this paper, SV, who is trained in the annotation of online patient conversations, provided a manual summary of each topic-specific document collection in the summarization corpus. With respect to symptom-related document collections, the annotator was instructed to focus on summarizing the impact of symptoms on patients’ daily functioning and quality of life. Regarding treatments, the goal was to summarize outcomes of the treatment as well as side effects or any other aspect related to the experience with these treatments. Consequently, the gold standard comprised 127 manual summaries of the symptoms and treatments listed in Multimedia Appendix 1. Given the substantial effort required in creating such summaries (approximately 3.5 hours per summary, >400 hours in total), it was not feasible to summarize each set of documents by several annotators. We discuss this further in the *Discussion* section while highlighting limitations.

The manual summary for the *rash* symptom, for instance, was as follows:

Patients perceive rashes as being horrible and uncomfortable, yet common. They are a great impairment for many patients since they are often painful and itchy. Some patients report that they cover multiple parts of the body, while others report it being localized to body parts, such as the face or the breast. They also report on the color and feel of the rash: pink, red, photosensitive, raised, large, hive-like, warm, etc. The appearance of the rashes changes with time, for some patients it became better (e.g. less itching), while for others worse (e.g. redder and hotter). Rashes can have different causes: they can be the side effect of treatments, such as chemotherapy, allergic reactions or symptoms. The cause for the rash is often unclear among patients, but it is often said that usually a rash is just a rash.

For the texts to fit the input size of the LLMs used in the study, the length was truncated to 1024 tokens. From the 127 manually generated reference summaries, 3 (2.4%) were used as examples for few-shot training, whereas the remaining 124 (97.6%) were used for evaluation only. For the fine-tuning experiments, the dataset was divided into 3 nonoverlapping sections: train, validation, and test. The resulting data sizes followed an 80:10:10 ratio, resulting in 79.5% (101/127), 9.5% (12/127) of the summaries for the train, validation, and test portions, respectively.

### Models Used for Patient Comment Summarization

For the task of summarizing texts, we relied on pretrained LLMs. On the one hand, we relied on encoder-decoder architectures (Flan-T5), as well as on decoder-only architectures (GPT models). As our focus lay on the experimental testing and comparison of different prompting techniques (instructions in the form of natural language), we used LLMs that have the capability to follow textual instructions. Flan-T5 and the family of GPT-3 models have this property as they have been trained on data to generate answers for instruction-like prompts using reinforcement learning, a process known as alignment [24]. We relied on the following 3 models in particular: Flan-T5-base, GPT-3, and GPT-3.5. Flan-T5 is an enhanced version of T5 that has been fine-tuned on a mixture of tasks using the Flan collection of datasets [25]. For this study, the Flan-T5 base model was used along with GPT-3 and GPT-3.5. GPT-3 is an advanced iteration of the GPT model that operates as an auto-regressive LM featuring an impressive 175 billion parameters [26]. GPT-3.5 is a refined version of its predecessor and boasts an enhanced ability to adapt to diverse prompts and instructions, making it a versatile tool for various applications. Together, these models are a good cross-section of the current LLM landscape and were well suited for our task.

### Prompting Techniques for Summarization

To explore the capabilities of LLMs to summarize patient comments, we compared various prompting strategies. We started with a prompt without examples or guidance on how to solve the task, called zero-shot prompting. As further methods, we enhanced the prompt by introducing examples of summarized patient comments (few-shot prompting); giving hints by DSP; and, finally, guiding the model by asking for intermediate results invoked by a set of self-defined questions (CoT prompting). By combining different techniques (few-shot prompting with DSP and zero-shot prompting with CoT prompting), we investigated the most effective prompting technique for summarizing patients’ comments.

One-shot prompting closely resembles the few-shot approach, differing in that a single demonstration is permitted along with the natural language task description [26]. The corresponding prompt template used can be found in Figure S1 in Multimedia Appendix 2.

To facilitate our experiment, we adopted this 1-shot prompt template. Within the template, (text) signifies the patient comments or experience regarding a symptom or treatment drawn from the example set, and (summary) represents the corresponding reference summary. Multimedia Appendix 2 contains other prompt templates that we exploited in our study.

Few-shot prompting introduces *k* examples consisting of a text and a summary provided by a human expert followed by the final text, which should be summarized by the model. For this study, *k* was set to 3 due to token limit constraints [26].

DSP is a framework that uses an adjustable LM to offer guidance to a black-box, frozen LLM to enhance its performance. Specifically, a policy LM is trained to generate discrete tokens acting as directional stimuli for each input of the LLM. These stimuli, such as keywords extracted from an article for summarization, are then integrated into the original input and fed into the LLM, steering its generation toward the intended target. DSP uses a customizable policy LM to produce the stimulus—in the case of summarization, keywords—to direct the LLM in generating a desired summary with improved metrics such as higher ROUGE scores or aligning more closely with human preferences. The policy LM can undergo training through supervised fine-tuning from annotated data and reinforcement learning from offline and online rewards, allowing for the exploration of directional stimuli that better align LLMs with human preferences. This adaptable framework is applicable to various LMs and tasks [27]. For this study, instead of a policy LM, KeyBERT [28] was used with the default parameters along with Keyphrase Vectorizers to extract accurate keywords for each patient comment that are given as hints to the summarizing LLM. KeyBERT is a straightforward and user-friendly approach to extracting keywords using Bidirectional Encoder Representations From Transformers (BERT) embeddings to generate keywords and key phrases that closely match the important content of a document [28]. Combining key phrase vectorizers with KeyBERT for key phrase extraction leads to a PatternRank approach, enabling the extraction of grammatically accurate key phrases that closely align with the document’s content. In this process, the vectorizer initially extracts candidate key phrases from text documents, and KeyBERT then ranks these candidates based on their similarity to the document. The top *n* most similar key phrases can be regarded as document keywords. The use of Keyphrase Vectorizers alongside KeyBERT offers the advantage of obtaining grammatically correct key phrases as opposed to simple *n*-grams of predefined lengths [29]. In this study, both keywords extracted using KeyBERT with Keyphrase Vectorizers and frequent terms occurring in the patient comment were combined to form a single set that was used as hints or directional stimuli for each patient comment to generate summaries. For example, the hints retrieved and used for the rash symptom were given as follows: severe rash; hives rash; rash hives; simple rash; red rash; cause; have; describe; impair; increase.

CoT prompting fosters reasoning capabilities by incorporating intermediate reasoning steps. The fundamental concept of this method is straightforward. By introducing phrases such as “Let’s think step by step” or a similar instruction, it triggers the model to reason step by step, outputting intermediate results that are combined with other intermediate results into an overall summary. In our study, CoT prompting for the patient comment summary generation was done based on the element-aware summarization approach in the zero-shot setting. In the element-aware summarization approach using CoT, as part of element extraction, we first asked the LLMs to extract important elements mentioned in the patient comment using the following manually set guiding questions: (1) What is the main health- or medical-related topic discussed in this patient comment? (2) What are the important topics mentioned in this patient comment? (3) What are the symptoms and diseases mentioned in this patient comment? (4) What are the recovery methods or treatments mentioned in this patient comment?

Next, we concatenated the extracted elements (ie, the answers to the aforementioned 4 questions [A]) with the following prompt (P)—“Let’s integrate the above information and summarize this patient comment:”—along with the patient comment (C) to prompt the LLM for patient comment summary generation. The input to the LLMs was [A; P; C], and the output was the final summary.

The advanced prompting techniques used in this study were applied in combination with each instruction-based model. Here, we tailored the use of these prompting techniques to the specific characteristics and insights derived from the data and task setup. For instance, with DSP, keywords extracted from patient comments were used as hints for generating summaries. Similarly, in CoT prompting, guiding questions were manually set to extract important elements from patient comments for summary generation. The prompt templates for summary generation, initially published by Google Research for Flan-T5 [30], were adopted as the baseline prompt template for implementing zero-shot, 1-shot, and few-shot prompting across all 3 models in our study. The same prompt template for different prompting techniques was followed for all 3 models except for CoT prompting. Together, this is a comprehensive collection of common prompting techniques, allowing for both a broad overview of the field as well as detailed insights into advantages and disadvantages of single techniques with respect to our task of summarizing patient experiences.

### Experimental Setup

For our experiments, we used the pretrained model versions of all the models (Flan-T5, GPT-3, and GPT-3.5), as shown in Table 1. The table also shows the decoding parameters that were used for all the models across all prompting techniques. For all the models, the summarization length was set to 300 tokens to balance efficiency and completeness while remaining reasonably close to the maximum token length of the ground-truth summaries (377 tokens). Following previous work [31], the temperature was set to 0.97 to limit the randomness of the generated text in combination with our sampling-based decoding strategy. High temperature values decrease the deterministic behavior of the text generation model. A value of 0.97 corresponds to a moderate level of randomness, representing a trade-off between avoiding repetitive and blank summaries and allowing for too much randomness and unfaithful generated texts.

**Table 1:** Pretrained model versions and decoding parameters of all the models used in the experiment.

Model	Pretrained model version	Decoding parameters
Flan-T5	flan-t5-base	min_length=150 max_length=300 do_sample=True Temperature=0.97 repetition_penalty=1.2
GPT-3	text-davinci-003	Temperature=0.97 max_tokens=300 top_p=0.9 presence_penalty=1
GPT-3.5	gpt-3.5-turbo-0301	Temperature=0.97 max_tokens=300 top_p=0.9 presence_penalty=1

To test the effect of fine-tuning, the Flan-T5 model was trained using the preprocessed train split of our dataset with the following training parameters: batch size of 1, a total of 10 training epochs, a learning rate of 0.00002, and weight decay of 0.01.

The chosen hyperparameters were set to fine-tune the Flan-T5 model effectively while minimizing the risk of overfitting. A small batch size helps manage memory limitations and adds implicit regularization. The 10 training epochs allow the model to learn from the data without excessive training, whereas the low learning rate (0.00002) ensures stable updates to pretrained weights, preventing drastic changes that could lead to overfitting. The weight decay of 0.01 acts as regularization by penalizing large weights, further preventing overfitting. In addition, by using the same decoding parameters for both fine-tuned and non–fine-tuned Flan-T5 models, consistency in evaluation was maintained. To prevent overfitting, the model’s performance was monitored on a validation set, ensuring that it did not memorize the training data.

The fine-tuned Flan-T5 model was used to generate the summaries with the same decoding parameters as shown in Table 1 for the non–fine-tuned Flan-T5 model.

Automatic summaries were generated for all models using the 7 prompting strategies. This was done for the 7 symptoms and 117 treatments as 1 symptom and 2 treatments were used as examples in the prompt in the few-shot cases, yielding a total of 2604 summaries (124 summaries per 7 prompting strategies for 3 models). In addition, for further experiments exploring the influence of fine-tuning, Flan-T5 was trained and validated on 113 summaries (n=101, 89.4% of the summaries for training and n=12, 10.6% of the summaries for validation) across 8 symptoms and 105 treatments. The remaining 11% (14/127) of the summaries for 14 treatments were used to measure performance in our automatic evaluation.

### Automatic Evaluation

To evaluate the generated texts automatically, we relied on state-of-the-art evaluation metrics used in text generation research, in particular, the token-based ROUGE and BERTScore. These metrics allowed us to compare the similarity of the generated texts to the manually created reference. The token-based ROUGE metrics essentially compute the overlap of phrases between the automatically generated summarization and the reference summarization, thus estimating the similarity of both summaries. BERTScore relies on embeddings and, thus, compares the summaries at a more semantic level, abstracting from the specific words or phrases that appear.

In this study, the generated summaries from all 3 models were evaluated using ROUGE, and the rescaled *F*_1_-score of BERTScore was calculated using the hidden representation states of the 17th layer of RoBERTa Large without inverted document frequency–weighting for the generated summaries. For ROUGE, we used 3 variants: ROUGE-1, ROUGE-2, and ROUGE-L. We used these 3 ROUGE variants to thoroughly evaluate the generated summaries. Each variant served a specific purpose: ROUGE-1 measures unigram (single-word) overlap to assess the occurrence of keywords without looking at the context, ROUGE-2 evaluates bigram (pair of consecutive words) overlap to capture context and word associations, and ROUGE-L analyzes the longest common subsequence to evaluate fluency and structural coherence [32]. These metrics collectively provide a nuanced assessment of the generated summary’s content, coherence, and adherence to the reference summary.

We present the average score considering all 124 test instances by computing the ROUGE and BERTScore values for each reference summary compared with the generated texts. A case study was conducted involving 2 summaries that were analyzed qualitatively and discussed in more detail, comparing the different generated versions of it in all model settings. In addition to the experiments with LLMs, we calculated the ROUGE and BERTScore values for a baseline using the concatenation of all input texts as a trivial summarization baseline that does not summarize anything.

### Ethical Considerations

Following recommendations on ethical methods to analyze data using NLP [33], the data were automatically anonymized by removing user IDs, personal names, addresses, and telephone numbers to minimize the risk of identification of a natural person to ensure compliance with the General Data Protection Regulation (GDPR). While the collected data still represent personal information, given the fact that we only analyzed data that had been made manifestly public by users without further provisions to protect the data, the use for research purposes can be argued to be acceptable given that there is a public interest in using these data to improve health care and that other means to collect such insights (eg, surveys) would represent a higher burden for patients [33]. On the other hand, the harm caused to patients by analyzing the data can be argued to be minimal as the content is already publicly available and no new information is released as part of the process.

According to the GDPR, there is a trade-off between the legitimate interests of data processors to process data that need to be balanced against the data protection rights of data subjects. In this particular case, there is a legitimate interest by society to rely on public data shared by patients as these data can contribute to improving the standard of care as well as the development of drugs that are relevant and address outcomes of relevance to patients. Furthermore, given that patients made their data “overtly and explicitly public” without any additional protection, we can assume that the sensitivity of the shared data is not that critical, and so exploiting them for the legitimate purpose of improving health care services is acceptable. The GDPR foresees that consent has to be obtained from data subjects to process their data. Given that the data were anonymized, patients cannot be directly contacted to obtain consent. In this respect, we processed the data making use of the exemption mentioned in the GDPR for processing data without explicit consent in case obtaining consent is not possible “with a reasonable effort.” Obtaining consent would, in our case, not represent a reasonable effort as each patient would have to be contacted individually, and some patients might not even be active anymore on the forums or sites in question. Contacting specific users would require registering on the platform in question, which in many cases would violate the terms and conditions of the platform as only patients are allowed to register. On many forums, direct messages cannot be sent to users, so they could not be directly contacted. In general, it has been a matter of debate whether observational studies can be ethical without patient consent and which conditions would need to be met for the absence of patient consent to be ethical. The United Kingdom’s Economic and Social Research Council, for instance, considers ethical the forgoing of patient consent as long as a study is not “undertaken lightly or routinely.” It is only justified if important issues are being addressed and if matters of social significance that cannot be uncovered in other ways are likely to be discovered [34]. A broader review of ethical recommendations [33] suggests that social media listening (SML) for health research can be ethical without informed consent in cases in which anonymization is ensured, harms are minimized, and there is a clear public benefit resulting from the analysis. As argued previously, all 3 criteria were fulfilled for our study.

## Results

### Overview

The evaluation was guided by three research questions: (1) Which model has the best performance compared to the reference summaries? (2) Which prompting techniques are most successful? (3) Does fine-tuning increase the models’ performance in generating patient comment summaries by only using a small amount of training data?

**Figure 1:**
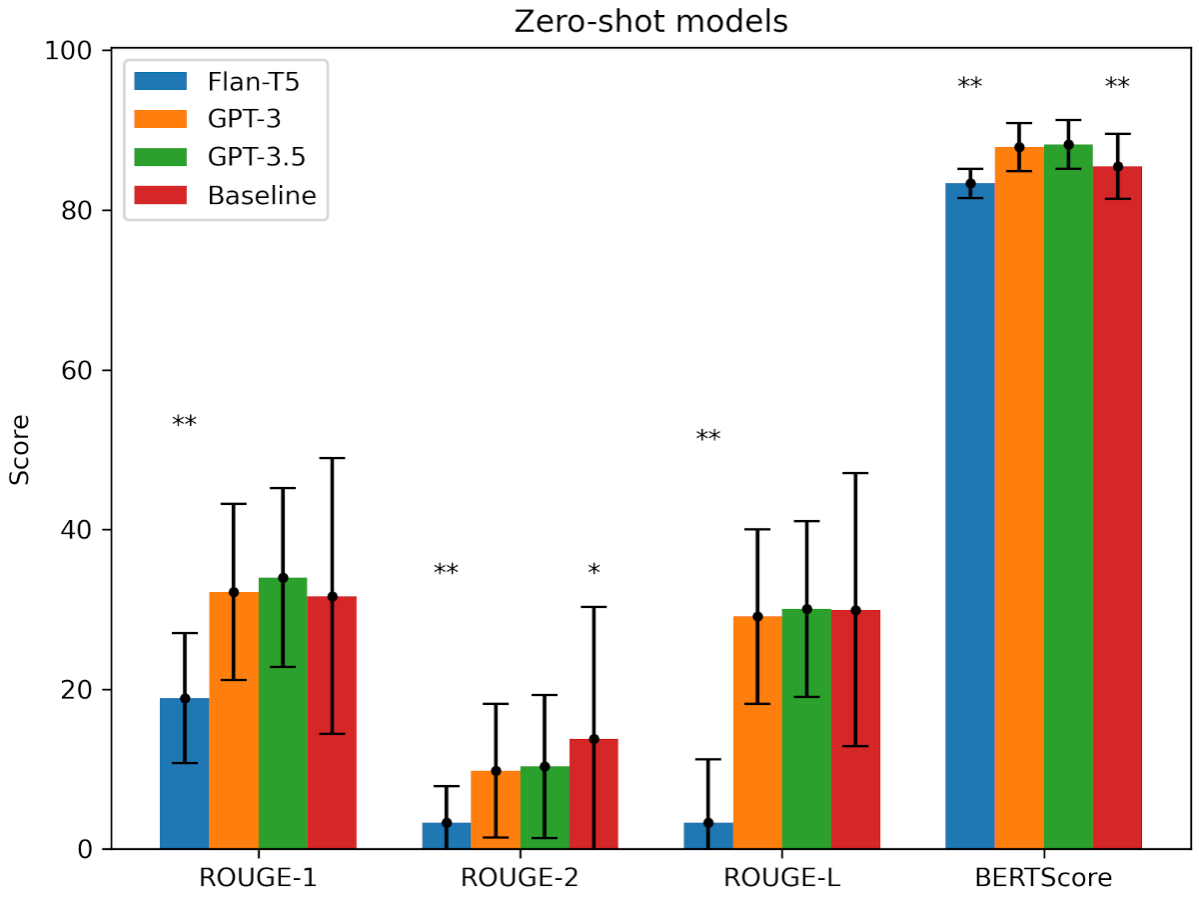
Mean scores per model and metric with corresponding SDs as error bars. Recall-Oriented Understudy for Gisting Evaluation (ROUGE) scores and BERTScore were obtained for all zero-shot models without fine-tuning; *P* values were obtained by comparing the mean scores for each model to the overall best-performing model (ie, Generative Pretrained Transformer [GPT], GPT-3.5). The figure shows that GPT-3.5 performed best for all metrics except ROUGE-2, for which the baseline performed best. *P<.05; **P<.001.

Regarding the performance of the different models, Figure 1 shows the average ROUGE and BERTScore values for 124 summaries generated using zero-shot prompting on 3 models. Flan-T5 performed worst for all metrics and with very high significance as well (*P*<.001).

In contrast, GPT-3.5 performed better than all the other models (including the baseline) for all metrics, with a margin ranging from 0.4% to 79.8% except for ROUGE-2, where the baseline performed significantly better (*P*<.05). GPT-3 in all cases performed slightly worse than GPT-3.5 but better than Flan-T5.

The average text length of the input text (and, hence, the output of the baseline) was 4199 (SD 7809) words, which was much higher than the average text length of the GPT-3.5–generated summaries, which was 446 (SD 214) words. Therefore, the count of bigrams was higher in general for the baseline when compared to the GPT-3.5 summaries due to its high average text length, which is not suitable for an actual summary. However, in contrast to the baseline, GPT-3.5 generated reader-friendly summaries that were shorter than the original text, having a higher semantic closeness to the reference than the baseline (+2.8 points in BERTScore). A *t* test was carried out comparing all other model variants to GPT-3.5 to confirm the significance of the results. The exact results can be found in Table S1 in Multimedia Appendix 2.

**Figure 2:**
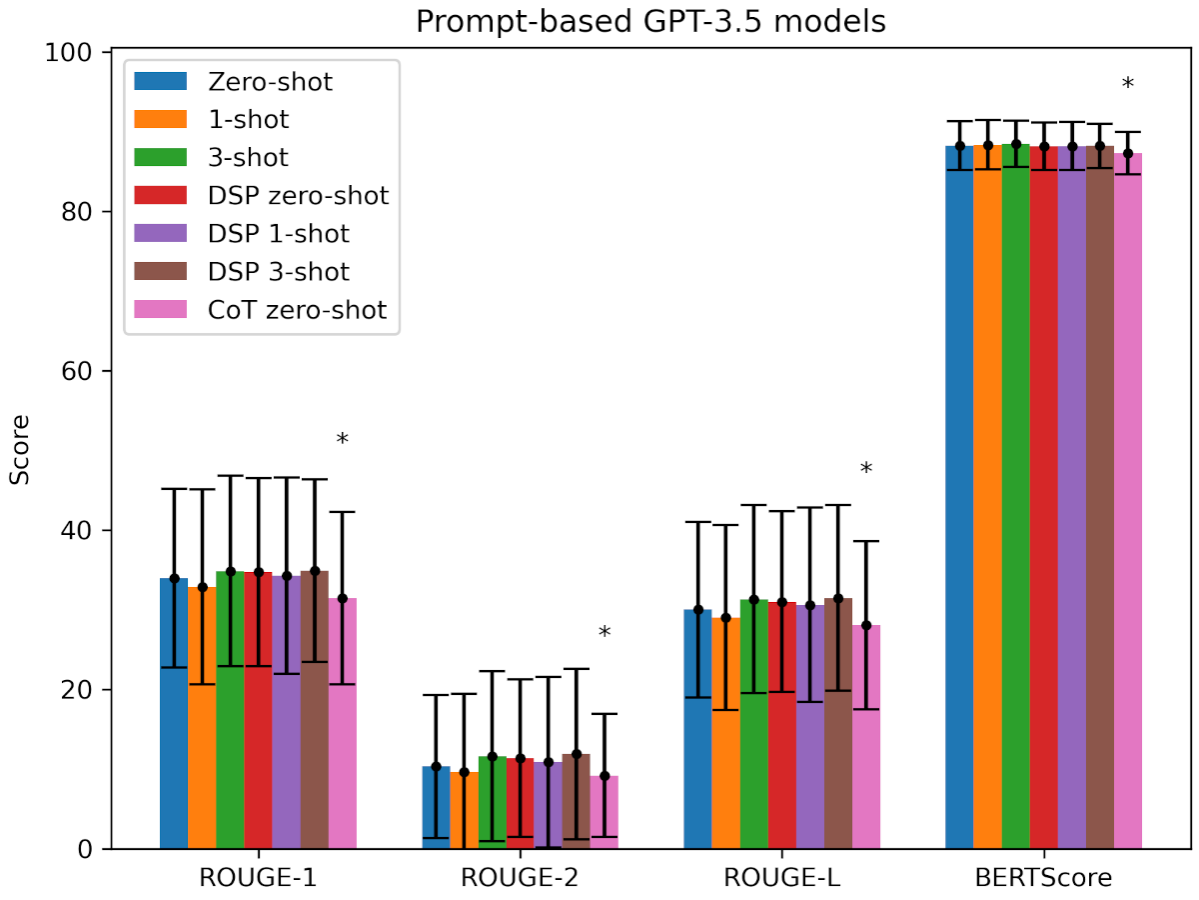
Mean scores per prompting strategy and metric for Generative Pretrained Transformer [GPT], GPT-3.5 with corresponding SDs as error bars—Recall-Oriented Understudy for Gisting Evaluation [ROUGE], ROUGE-1, ROUGE-2, ROUGE-L, and BERTScore scores for prompt-based GPT-3.5 models, with *P* values obtained by comparing all prompt-based GPT-3.5 models to the best-performing GPT-3.5 directional stimulus prompting (DSP) 3-shot model. The figure shows that the DSP 3-shot model performed best. However, none of the results was significant except for the worst performance of chain-of-thought (CoT) zero-shot training. *P<.05; **P<.001.

Focusing on GPT-3.5 as the best overall model for zero-shot prompting, we proceeded to investigate the impact of different prompting strategies on the summarization performance. Figure 2 shows the average ROUGE-1, ROUGE-2, ROUGE-L, and BERTScore values for 124 summaries generated using the 7 different proposed prompting techniques. Again, the exact results can be found in Table S2 in Multimedia Appendix 2. Regarding the ROUGE score, the obtained evaluation scores show that DSP using 3 examples outperformed other prompting techniques. For BERTScore, 3-shot prompting without DSP outperformed all the other models. However, the differences among the different prompting techniques regarding their performance were only minor and not significant in most cases, especially for BERTScore. The only significant (*P*<.05) result was that CoT zero-shot prompting performed worse than all other prompting techniques.

Multimedia Appendix 2 provides tables detailing the results of the 7 prompting strategies for the Flan-T5 and GPT-3 models.

**Figure 3:**
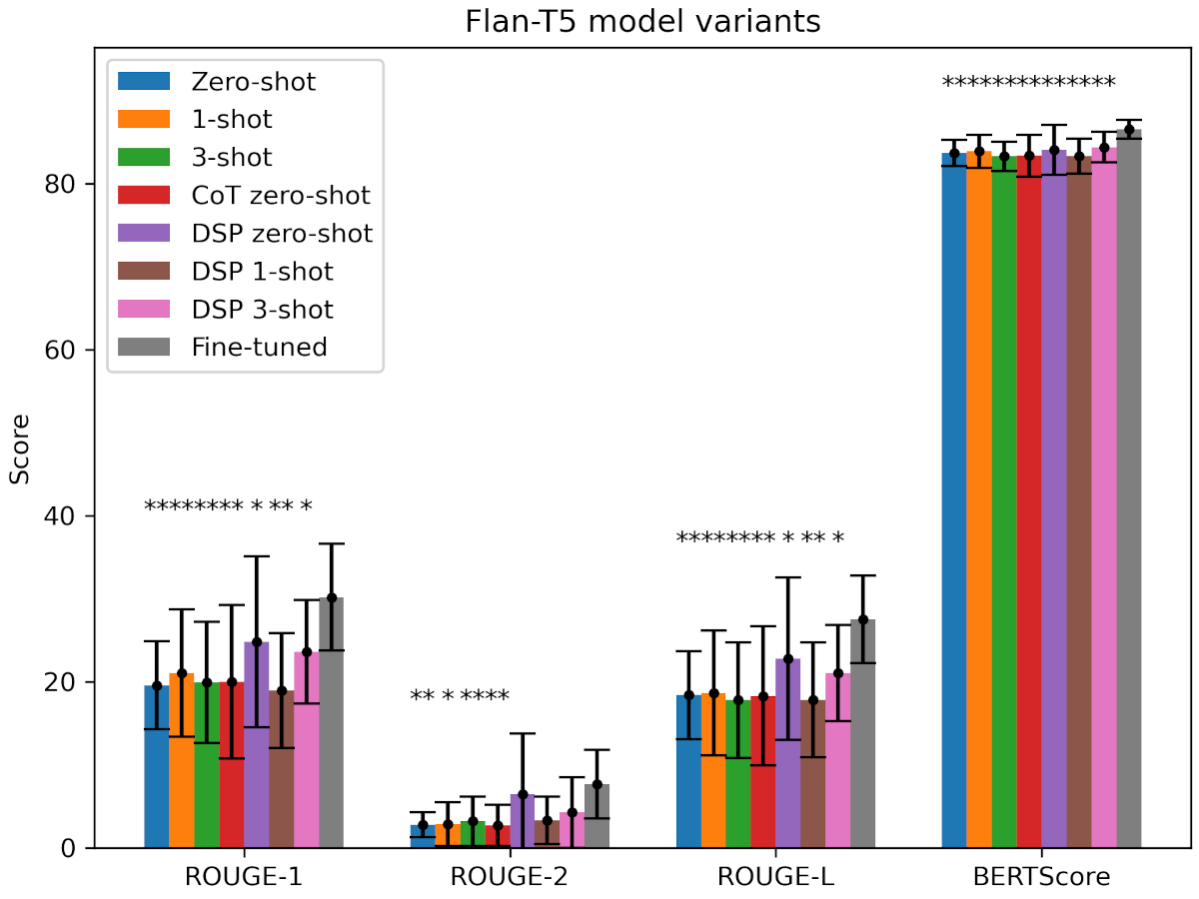
Mean scores per prompting strategy and model variant and metric for Flan-T5 with corresponding SDs as error bars—Recall-Oriented Understudy for Gisting Evaluation [ROUGE] ROUGE-1, ROUGE-2, ROUGE-L, and BERTScore scores for the Flan-T5 model variants, with *P* values obtained by comparing all Flan-T5 models to the fine-tuned Flan-T5 model. The figure shows that the fine-tuned model outperformed all non–fine-tuned models no matter what prompting strategy was used. *P<.05; **P<.001; CoT: chain of thought; DSP: directional stimulus prompting.

To explore the effect of fine-tuning under the restriction of having only a small amount of training data, Figure 3 shows the average ROUGE-1, ROUGE-2, ROUGE-L, and BERTScore values obtained for the fine-tuned Flan-T5 compared with the prompt-instructed Flan-T5 model for 14 test instances (we only considered Flan-T5 for fine-tuning as, given that GPT-3 and GPT-3.5 are closed source, they cannot be fine-tuned locally with a full control over the training process and involved data). Again, the exact results can be found in Table S3 in Multimedia Appendix 2. The fine-tuned Flan-T5 model outperformed the non–fine-tuned Flan-T5 model in all cases, with the non–fine-tuned variants performing worse with normal (*P*<.05) or even very significant (*P*<.001) results in almost all cases. Only for the ROUGE-2 scores of the 3 DSP strategies the results were not significant. Thus, upon evaluating the obtained scores, it is evident that fine-tuning—even with only a small amount of training data—is more effective than any prompting strategy for the Flan-T5-base model.

It is important to note that the fine-tuned Flan-T5 model cannot be directly compared to the prompt-based GPT models; it can only be compared to the prompt-based or non–fine-tuned Flan-T5 models, as shown in Figure 3.

All in all, among the tested models, GPT-3.5 performed best. The best-performing prompting strategy was DSP 3-shot prompting for the ROUGE scores and 3-shot prompting for BERTScore. For Flan-T5, the fine-tuned version performed better than all prompting strategies on non–fine-tuned Flan-T5 models.

### Case Study

To discuss the performance of the approach in a more qualitative manner, we analyzed the different summaries provided by the different models in comparison to the reference summary for 1 treatment (Carboplatin) and 1 symptom or phenotype (neoplasm). We selected examples in which the ROUGE scores were highest. While it might seem unfair to select the topics with the best results, the case study is not meant as a proper evaluation but only to provide examples of how the summaries differed across methods.

Textbox 1 shows the different summaries of the patient experience with Carboplatin, including the reference summary and the GPT-3.5 zero-shot, GPT-3.5 three-shot, and GPT-3.5 DSP 3-shot summaries. The reference summary emphasizes that Carboplatin, being a new treatment and a good option to treat triple negative breast cancer, has good study results and better tolerability and is becoming increasingly popular. It emphasizes the side effects, such as hair loss and heart damage. It mentions that side effects might increase in combination with other drugs, such as Taxol and Gemcitabine.

The GPT-3.5 zero-shot summary highlights the efficacy (although not a good study performance explicitly), side effects (hair loss but also hearing loss and tinnitus), and popularity of the treatment. In addition, it highlights better tolerability of Carboplatin and Taxol in comparison to Carboplatin alone. It provides further details on dosage and scheduling and even goes as far as talking about the mechanism of action.

The GPT-3.5 three-shot summary highlights that Carboplatin has had good study results, as well as the side effects, mentioning hearing loss and tinnitus. The summary misses heart damage but includes an additional side effect not mentioned in the reference summary (ie, tinnitus). The summary goes further than the human-generated summary in terms of discussing the regimen and scheduling in addition to discussing outcomes in combination with other drugs.

The GPT-3.5 DSP 3-shot summary emphasizes the side effects (again, hearing loss and tinnitus) as well as better tolerability. It highlights that Carboplatin has shown promising results in clinical trials. Beyond that, it provides more detail on how patients deal with the side effects by using other treatments.

Overall, the 3 summaries cover the most important points highlighted by the human reference summary, providing some additional details (eg, dosage, mechanisms of action, other side effects, and combination with other drugs or treatments to reduce side effects).

Textbox 2 shows different summaries of the patient experience with neoplasm, including the reference summary and the GPT-3.5 zero-shot, GPT-3.5 three-shot, and GPT-3.5 DSP 3-shot summaries. The reference summary highlights patients’ experiences with neoplasm, focusing on tumor size, aggressiveness, and growth rate. It discusses tumor structure, including receptors and mutations and varied treatment responses. Patients reported problems such as fluid retention; lymphedema; tissue damage; severe pain; and complications from spinal spread, such as vertebral fractures and bone marrow impairment. Treatment outcomes varied, with some tumors decreasing, staying stable, or growing despite interventions.

The GPT-3.5 zero-shot summary discusses patient experiences with neoplasms, covering tumor characteristics, treatment options, and responses. It distinguishes between benign and malignant tumors and mentions the potential for immunotherapy. However, it lacks details on specific patient-reported problems and complications highlighted in the reference summary, such as fluid retention and bone marrow impairment due to spinal spread.

The GPT-3.5 three-shot summary echoes the reference summary’s emphasis on patients’ experiences with tumor characteristics, including size, grade, and aggressiveness. It acknowledges the importance of postsurgical treatments such as chemotherapy and radiation in preventing cancer recurrence. It also discusses tumor markers and lymph node involvement. However, it provides a more concise overview, focusing on patients’ concerns about tumor growth and aggressiveness and the importance of treatment in improving survival rates.

The GPT-3.5 DSP 3-shot summary delves into patients’ tumor descriptions, covering size, stage, grade, and lymph node involvement. It acknowledges larger, aggressive tumors necessitating postsurgical treatments such as chemotherapy or radiotherapy. In addition, it recognizes benign tumors and the significance of tumor markers such as estrogen receptor, progesterone receptor, and HER2 in treatment decisions. The summary also discusses tumor flare after biopsy and size fluctuations over time, stressing the uniqueness of each patient’s tumor and the need for personalized treatment plans, echoing the reference summary’s focus on varied tumor responses and patient challenges.

Overall, the 3 summaries cover the key points highlighted in the reference summary while providing additional details such as genetic mutations, treatment responses, and the importance of individualized treatment plans.

**Reference summary, GPT-3.5 zero-shot summary, GPT-3.5 three-shot summary, and GPT-3.5 directional stimulus prompting (DSP) 3-shot summary about Carboplatin. The textbox shows that all summaries mention the most important points.**

**Reference summary**

- “Carboplatin is a more recent treatment, deemed by patients as a good option to treat TNBC, having encouraging results in studies and a better tolerability. The treatment is more novel, yet becoming more popular. But it also causes side effects such as hair loss, heart damage. When combined with other drugs such as Taxol and Gemcitabine, the side effects increase.”

**GPT-3.5 zero-shot summary**

- “The patient comment covers a variety of topics related to Carboplatin, a chemotherapy drug used to treat Triple Negative Breast Cancer (TNBC). The comment discusses the efficacy of Carboplatin in treating TNBC even without HER2 positivity, potential side effects such as hearing loss and tinnitus, and its popularity as a treatment option. The comment also touches on the dosing schedule and suggests that weekly carbo taxol is easier to tolerate than every 2 weeks IV carbo. Some comments mention hair loss and other side effects associated with Carboplatin. The comment also highlights the role of calcium carbonate in reducing the absorption of other medicines from the gut.”

**GPT-3.5 three-shot summary**

- “Carboplatin is a chemotherapy drug used to treat Triple Negative Breast Cancer (TNBC), even if the patient does not have Her 2 positivity. Patients report that Carboplatin has been shown to have good results in trials and may be a better option than other treatments such as AC. However, some patients also report potential side effects of Carboplatin, such as hearing loss and tinnitus. Carboplatin can be given as a weekly regime or every two weeks, but the latter may cause a decrease in hemoglobin levels. Despite this, Carboplatin is still seen as an effective treatment option for high-grade TNBC. There is also ongoing research into the use of Carboplatin with other drugs to improve treatment outcomes.”

**GPT-3.5 DSP 3-shot summary**

- “Carboplatin is a commonly used chemotherapy drug for treating TNBC, although it can cause side effects such as hearing loss and tinnitus. Patients report mixed experiences with the drug, with some finding it tolerable and others experiencing difficulty with its side effects. Carboplatin has shown promising results in clinical trials and is being studied in combination with other agents such as leronlimab. It is not clear why some patients are prescribed Carboplatin over other chemotherapy drugs, but it may be preferred for high-grade TNBC or in combination with radiotherapy. Patients also report using bicarbonate and calcium carbonate to manage side effects and improve blood pH levels during treatment.”

**Reference summary, GPT-3.5 zero-shot summary, GPT-3.5 three-shot summary, and GPT-3.5 directional stimulus prompting (DSP) 3-shot summary about neoplasm. The textbox shows that, again, all important details are mentioned in the summaries, such as genetic mutations and treatment responses.**

**Reference summary**

- “Regarding neoplasm, patients often describe the size of their tumors, how aggressive they are and how fast they grow. Furthermore, they also describe their structure, which receptors, mutations they have and how fast or slow they respond to treatments. There were many varied responses in these regards, e.g. many small but also many large treatments, responsive and non-responsive treatments and different types of tumors. Neoplasms caused an array of problems for patients such as fluid retention, lymphedema, acidity, tissue dama, a concave chest after removal of the tumor and chest muscle, inflammation, compression of nerves, severe pain and an immunosuppressive environment. For some patients where it spread to their spine, it impaired their bone marrow, caused complications, broke vertebrae and aggravated nodes. Some neoplasms decreased with treatment, but others either decreased and then came back, stayed stable or even grew despite treatment.”

**GPT-3.5 zero-shot summary**

- “The patient comments mention various sizes and stages of tumors, as well as the presence or absence of lymph node involvement and markers such as HER2. Some tumors are benign while others are malignant, and treatment options include surgery, chemotherapy, and radiotherapy. Response to treatment varies, with some tumors showing complete response and others being slow-growing. In some cases, genetic mutations may be acquired under the selective pressure of therapy, and hot tumors with inflammation may respond to immunotherapy. Some tumors contain a mix of different cell types, and there may be concordance or discordance between cell-free DNA and tissue samples. Overall, the comments suggest that cancer diagnosis and treatment can be complex and individualized, and it is important to trust medical professionals while seeking support from others who have gone through similar experiences.”

**GPT-3.5 three-shot summary**

- “Patients share their experiences with tumors, including their size, grade, and aggressiveness. They also mention the importance of postsurgical treatments, such as chemotherapy and radiation, in preventing cancer recurrence and improving survival rates. Patients highlight the difference between benign and malignant tumors and share their tumor markers and lymph node involvement. Some patients express concern about the growth and aggressiveness of their tumors, while others report slow-growing tumors.”

**GPT-3.5 DSP 3-shot summary**

- “Patients describe their tumors in terms of size, stage, grade, and involvement of lymph nodes. Some patients report having larger and more aggressive tumors, which require postsurgical treatments such as chemo or radiotherapy to manage them. However, some tumors are benign and do not necessarily indicate cancer. Patients may also have a combination of different tumor markers such as ER, PR, and HER2, which may affect the aggressiveness of the cancer and the recommended treatment options. Tumor flare is a real phenomenon that can occur after biopsy, and tumor size can fluctuate over time. Some patients also report elevated tumor markers in their blood. Ultimately, each patient’s tumor is unique and requires an individualized treatment plan.”

## Discussion

### Principal Findings

Our evaluations of different models on the task of generating summaries about the patient experience revealed that GPT-3.5 was the best-performing model. This is no surprise as it is the model with the most parameters (approximately 375 billion) of the set of models examined. GPT-3 has 175 billion parameters, and the smallest model was Flan-T5 with 0.2 billion parameters in the version we used. As the training data sources for the GPT models are not made public, we cannot attribute the differences in performance to differences in the training data.

Taking GPT-3.5 as the best-performing model (see research question 1), we examined in detail the impact of different prompting strategies. For the 3 ROUGE metrics, DSP 3-shot prompting emerged as the best strategy, whereas few-shot prompting with 3 examples received the best scores according to BERTScore (see research question 2). In any case, the results clearly show that providing at least 3 examples helps the model generate more tailored summaries. DSP 3-shot prompting benefits particularly from the fact that key relevant topics are provided as guidance to the model, which helps generate better summaries.

Our fine-tuning experiments with Flan-T5 showed that fine-tuning can have a positive effect in principle (see research question 3). However, we assume that the amount of data was still too low. Therefore, the fine-tuned Flan-T5 setting was not really comparable to the other settings that were not fine-tuned with a comparable number of examples. In particular, the results of Flan-T5 were, irrespective of fine-tuning, quite low compared to those of the other models. However, the results show the potential of fine-tuning within a particular model class.

Our case study using GPT-3.5 as the best-performing model with the best prompting strategies showed that the summaries produced by GPT-3.5 are reasonable, covering most of the aspects of the manually generated summaries while including some additional details or background. Our results demonstrated that such models can be used to speed up qualitative research by being able to summarize large amounts of text automatically. In addition, we confirmed a positive correlation between the quality of the summaries and the models’ size on the one hand and fine-tuning on the other hand. In March 2023, OpenAI released GPT-4, as well as the possibility to fine-tune models using a fee-based application programming interface. These new developments are promising and should be investigated further, yet they were not available at the time the experiments described in this paper were carried out.

Taken together, our results suggest that state-of-the-art pretrained LLMs can be a valuable tool to provide qualitative insights about the patient experience to better understand unmet needs, patient priorities, and how a disease impacts daily functioning and quality of life to inform processes aimed at improving health care delivery and ensure that drug development focuses more on the actual priorities and unmet needs of patients. In particular, the insights would allow for the focus of drug development and clinical development to be on outcomes that are relevant to patients and have the potential to substantially improve their quality of life. The insights provided by our method could be used to develop a more detailed understanding of patient journeys as a basis to improve patient care by focusing on the actual problems and unmet needs that patients face in different phases of their journey. The insights could further help in developing support programs that provide relevant information to patients when they need it to empower them to make better decisions. Finally, such qualitative insights could also inform the design and conceptualization of digital apps for patients.

As a notable limitation of this study, our manually created test set for the quantitative evaluation of the different approaches had a limited sample size. Although we presented an experiment with a fine-tuning approach, after splitting the small dataset into training, development, and test sets, we noticed that each split was too small to derive representative results for each setting. Having a larger dataset (at least by 1 order of magnitude) would enable more experiments with respect to fine-tuning. While the sample size was small, it is a remarkable result that fine-tuning had a positive impact on the task. A larger dataset would possibly have yielded even stronger results. However, as the focus of this study was on experimenting with (unsupervised) prompting approaches, we keep the experiments with a larger dataset for future work. A further important limitation is the fact that the reference summaries were created by only 1 annotator, which might bias the results to the perspective of this single annotator. The huge effort involved in creating these summaries did not make it possible to have multiple annotators providing the summaries. A larger dataset could be obtained via crowdsourcing platforms such as CrowdFlower or Prolific; however, a drawback of these platforms is that we cannot reliably control for the (domain) expertise of the labelers.

The summaries could not be directly compared using standard annotation metrics such as the Cohen κ but could have been compared in terms of ROUGE to each other to have an upper baseline for the scores.

Another limitation arises from the nature of our applied automatic metrics. The average text length of the output of the baseline approach (mirroring its input) was substantially higher (contradicting the idea of a summary that should be concise) than that of the GPT-3.5–generated summaries. However, in terms of our automatic metrics, this discrepancy in text length impacted the count of bigrams, favoring the baseline compared to the GPT-3.5 zero-shot summary. The automatic evaluation metric we used, for example, ROUGE-2, does not consider the text length, which may bias the results toward the longer text. Thus, while the baseline appeared to perform better according to ROUGE-2, it is important to interpret these results cautiously considering the difference in text length.

We compared the different LLMs and the different prompting strategies on our own dataset consisting of 127 reference summaries. The dataset might not be completely representative of all sorts of summarization tasks, possibly limiting the generalizability of the results to other related tasks. The comparison of the different methods and the results obtained are specific to the particular summarization task considered. It is an open question whether the results generalize to other summarization tasks. However, our focus was on determining whether LLMs can reliably and accurately summarize the experience of patients as shown on online posts. A question for further research is to better understand why the performance of models differs and which underlying factors are responsible for the differences. Understanding whether the differences in ROUGE and other scores represent semantically meaningful differences is also an important question for future work. Indeed, future work might strive for a more semantic assessment of the generated summaries by manual coders who could rate the generated text in terms of variables such as clarity, comprehensiveness, completeness, and readability.

Beyond this, since the time our experiments were carried out, newer LLMs have been released, in particular also GPT-4 and GPT-4o, which could also be tested. Other open-source models such as Llama could also be investigated.

To the best of our knowledge, our dataset is the only dataset consisting of manually created summaries of patient experience. Thus, we cannot evaluate our method on existing datasets. To allow researchers to compare their methods on our dataset, we will provide the dataset to anyone interested upon request.

Beyond improving the methodology, data basis, and models used, future research should show that the insights derived via summarization methods can indeed impact our understanding of what unmet needs and priorities patients have and positively impact drug development and optimization of patient journeys. This question is clearly out of the scope of this study but could be pursued in follow-up work. While we focused only on posts from patients with breast cancer, we see no reason why this approach could not generalize to other patient populations. However, this needs to be validated in future work.

### Related Work

We briefly discuss related work on using LLMs in health care in general, deep diving into work on using NLP for analyzing patient experiences on social media. We discuss the application of summarization in health care as well.

### LLMs in Health Care

Very recently, LLMs have been considered for different tasks and use cases in health care. In a recent overview paper, Clusmann et al [35] described the main areas in which there is the potential to apply LLMs in health care: research, patient care, and education. The use of LLMs to summarize patient discussions falls into the area of patient care as it has the potential to uncover unmet needs of patients that can contribute to improve drug development and patient care.

Beyond text summarization tasks, LLMs have also been investigated on use cases related to drug discovery [36] and have been tested on tasks such as helping discover new targets as well as predicting characteristics of drugs, including molecular pharmacodynamics, pharmacokinetics, and toxicity [37]. They have also been shown to be able to support the discovery of drug-drug interactions [38]. A recent review has even highlighted the opportunities, limitations, and barriers regarding the use of LLMs in medical diagnosis [39].

### LLMs for SML

SML has been established as a mature methodology to extract key insights about the patient experience as a way to inform drug development and improvement of patient care. A recent review analyzed 63 publications that performed SML [9]. Of the 63 publications analyzed, 38 carried out some computer-assisted analysis. The methods used covered a wide range of NLP methods to detect topics and concepts within textual data. While some published research used keywords and dictionaries to identify topics in some cases [40], other methods comprised binary matrix factorization, Bayesian estimation, and Latent Dirichlet Allocation [41]. Several of the reviewed publications stated that custom-trained NLP algorithms were used [42,43], and neural networks were also reportedly used to identify certain topics or expressions [44]. A total of 25 out of the 63 publications covered in the aforementioned review performed manual analysis, relying on the manual coding of small data samples. The methods that analyze social media posts automatically by using NLP typically extract certain predefined aspects of the patient experience, including key symptoms, their severity, and experiences with treatments. While they support the scalable analysis of large amounts of text, they are typically not suited to analyze open topics, nor do they provide detailed qualitative insights. Manual analysis methods that rely on the development of a specific coding can be used to answer very specific research questions from a qualitative perspective. However, they are not designed to scale and are necessarily limited to analyzing a smaller sample. The method proposed in this paper can be understood as a sweet spot between these 2 methodological ends of the spectrum. The automatic summary of patient experience conversations allows for the scaling to larger amounts of data and text while also allowing for the succinct summarization of the data to support qualitative understanding.

### Automatic Text Summarization in Health Care Applications

Summarizing texts is an important field in the area of NLP, aiming to condense a verbose text into a shorter version while preserving the essence of its meaning. While previous research started with extractive summarization (ie, selecting fragments from the text to be summarized that are concatenated to yield a summary), with the rise of LLMs, research has shifted toward abstractive summarization, in which a model generates a text instead of copying text fragments [45], resulting in a fluent text that helps the reader obtain an overview with minimal time investment. To this end, the input text is encoded into an internal representation that is enrolled by a decoder into natural language. By massive pretraining of LLMs [14], fine-tuning models toward summarization [12], or tailoring them toward instructions asking for summarizations [13], this task is becoming a deeply explored field in NLP.

In the domain of health care, automatic summarization of patient data has emerged as a vital area of research. In this section, we explore the recent advancements in summarization techniques of patient data and other types of data, highlighting the models and methodologies used in alignment with our research.

It has been previously investigated how LLMs can support tasks related to medical documentation and information summarization. LLMs have been particularly evaluated on the summarization of radiology reports, patient questions, progress notes, and doctor-patient dialogue [46]. The objectives of the study by Van Veen et al [46] were similar to those of ours in that the goal was to evaluate the effectiveness of LLMs on the task of summarizing clinical text. In contrast to our focus on patient experience reports from social media or patient forums, their work focused on other types of data (radiology reports, patient questions, progress notes, and dialogues). They used similar metrics for the evaluation of the automatically generated summaries compared to manual summaries (ie, ROUGE-L and BERTScore in addition to Bilingual Evaluation Understudy (BLEU). While they also examined similar models—Flan-T5 as well as GPT (using version 4 instead of 3.5)—they did not examine an exhaustive set of prompting strategies as we did. They also concluded that GPT had the best performance, generating summaries that were more complete on average than human summaries. These results are in line with the results of our study.

A recent study testing the ability to summarize medical evidence using LLMs has shown clear limits of these technologies as factual inconsistencies produced by LLMs are critical in some applications such as summarizing medical evidence, which requires increased care and accuracy as such results might inform the decision-making of many [47]. In their study, Tang et al [47] focused on the task of medical evidence summarization, referring to the process of extracting and synthesizing key information from a large number of medical research studies and clinical trials into a concise and comprehensive summary. They also used automatic evaluation metrics, including ROUGE-L, BLEU, and METERO to evaluate the automatically generated texts with respect to expert-created Cochrane reviews. They explored models from the GPT family only (GPT-3.5 and ChatGPT) but did not analyze the impact of different prompting strategies, relying only on zero-shot prompting. In terms of ROUGE-L scores, GPT-3.5 performed better than ChatGPT, although this was not the case for other metrics.

A study by Pal et al [48] investigated the effectiveness of various neural network architectures in generating hospital discharge summaries from electronic health records within the health care domain. They applied different models (BART, Longformer, T5, and Flan-T5) and examined the impact of fine-tuning on the results, relying on similar automatic evaluation metrics to those we used (ROUGE-1, ROUGE-2, and ROUGE-L). In contrast to our work, they did not use any instruction-following models from the GPT family, so they could not investigate the impact of different prompting strategies. In line with our results, their study showed that fine-tuning can consistently improve results.

Another research study by Nair et al [49] proposed a multistage approach that leveraged few-shot prompting techniques to generate summaries of patient-provider dialogues within the health care domain. They relied on GPT-3 as a model for their multistage approach and validated their method with respect to 100 clinical encounters. They examined in particular the impact of the number of examples provided in the prompt, varying among 1, 3, and 5 examples.

The effectiveness of pre-trained models for text summarization is further supported by the work by Ay et al [50]. Their work demonstrates the successful application of the T5 model pretrained on news data for abstractive text summarization of Turkish text documents. In contrast to our work, they did not focus on summarization of health-related texts and only relied on 1 sequence-to-sequence model, T5. In contrast, we also examined auto-regressive models from the GPT family and investigated the impact of different prompting strategies.

The study by Wang et al [51] introduced a novel element-aware summarization approach using GPT-3 and CoT prompting for abstractive text summarization. While they worked on a completely different domain (news texts rather than health-related texts), they followed a similar methodology, relying on the same evaluation metrics (ROUGE-1, ROUGE-2, and ROUGE-L as well as BERTScore), also examining the impact of fine-tuning BART and T5 compared to using GPT-3 with zero-shot prompting. Their results were mixed depending on the dataset. As a general trend, they showed that the larger models such as GPT-3 performed better than the smaller (but fine-tuned) models. This study is the only one in addition to ours investigating more advanced prompting strategies such as CoT.

Li et al [27] introduced DSP, which we used in our experiments, as a method to guide LLMs for text summarization tasks. They evaluated their proposed method on the task of summarizing news and also evaluated the suitability of the generated summaries using BLEU, ROUGE, Metric for Evaluation of Translation With Explicit Ordering (METEOR), and BERTScore. In terms of models, they also focused on instruction-following models such as GPT-3, InstructGPT, and ChatGPT. They showed that all the models can benefit from the use of directional stimuli, which is in line with our results.

Taken together, we see that none of the existing works has actually focused on summarizing patient reports from social media or forums. Most studies have also used automated metrics such as ROUGE, BLEU, METEOR, and BERTScore as we did, in some cases complementing this with a manual evaluation by experts. In terms of the models investigated, our study is similar to others in the sense that, in most studies, encoder- or decoder-based sequence-to-sequence models were investigated, including T5 and Flan-T5 in particular, as well as auto-regressive pretrained decoder-only models from the GPT family (GPT-3, GPT-3.5, GPT-4, and ChatGPT). In line with our results, most studies showed the superiority of the GPT family models. Our work is the only one that has investigated the impact of very different prompting strategies, showing in particular the impact of using direct response stimuli in combination with few-shot training. In terms of comparison, it is not possible to directly compare our results to those of other studies as none of the aforementioned studies focused on patient reports.

### Conclusions

Exchanging information with peers on social media sites, support forums, or social networks, as well as receiving emotional support from them, is important for patients in coping with their conditions and being empowered. However, the information they share on public sites is unstructured and vast, not accessible for those interested in using it to improve health care. Methodologies for generating qualitative information at a large scale from these datasets have so far been missing.

Toward closing this gap, this study shows that it is possible to accurately and reliably summarize the content shared by patients on social media sites, social networking sites, discussion forums, and support groups automatically. Our study explored the feasibility of automatically summarizing experiences shared by patients online using generative LLMs combined with different prompting strategies. Our comparative study highlights the effectiveness of different advanced prompting techniques used with generative LMs in summarizing online patient comments. Notably, few-shot models, particularly the 3-shot variants, exhibited superior results compared to their zero-shot and 1-shot counterparts. Within the few-shot category, GPT-3.5 stood out with higher evaluation scores compared to those of the other models. In the context of DSP models, DSP few-shot (3-shot) models, specifically GPT-3.5, delivered the most favorable results. Upon comprehensive comparison, DSP-based 3-shot models emerged as the most efficient prompt-based models for summarization tasks, especially in the context of patient comment summarization. Although our experiments fine-tuning the smaller model Flan-T5 increased its performance by 50% in terms of the longest subsequence common to the reference, our advanced prompting techniques using larger models such as GPT-3.5 were superior in the explored domain.

Our results open up the possibility of using the qualitative insights generated at a larger scale to inform processes of drug development, design of clinical trials, development of patient journeys, public policy making, and informing the development of digital health apps. Information about unmet needs; symptoms; and their impact on the quality of life of patients, disease burden, and experiences with existing treatments provides valuable insights to inform the aforementioned processes and, ultimately, improve the state of care for patients.

## Appendix

Multimedia Appendix 1 List of frequent symptoms and treatments mentioned in the dataset, numbered from 1 to 127.

Multimedia Appendix 2 Additional results and listings.

## Abbreviations

BERT: Bidirectional Encoder Representations From Transformers
BLEU: Bilingual Evaluation Understudy
CoT: chain of thought
DSP: directional stimulus prompting
GDPR: General Data Protection Regulation
GPT: Generative Pretrained Transformer
LLM: large language model
LM: language model
METEOR: Metric for Evaluation of Translation With Explicit Ordering
NLP: natural language processing
ROUGE: Recall-Oriented Understudy for Gisting Evaluation
SML: social media listening
