# Supplementary material for: Summarizing Online Patient Conversations Using Generative Language Models: Experimental and Comparative Study
Source: JMIR Med Inform. 2025 Apr 14;13:e62909. doi: 10.2196/62909 (PMC12038288; doi:10.2196/62909)
Supplement: Multimedia Appendix 1 [file medinform_v13i1e62909_app1.ocx]

List of frequent symptoms and treatments mentioned in the dataset, numbered from 1 to 127.

**Symptoms**

1. Rash
2. Breast pain
3. Nipple discharge
4. Seborrheic dermatitis
5. Inverted nipple
6. Erythema
7. Edema
8. Neoplasm

**Treatments**

9. Abemaciclib

10. Abraxane

11. Anastrozole

12. Carboplatin

13. Clinical nutrition

14. Capecitabine

15. Capivasertib

16. Cryosurgery

17. Cyclophosphamide

18. Cytoxan

19. Chemotherapy

20. Acupuncture

21. Adriamycin

22. Afinitor

23. Alpelisib

24. Aranesp

25. Aredia

26. Arimidex

27. Aromasin

28. Aromatherapy

29. Atezolizumab

30. Avastin

31. Bevacizumab

32. Breast reconstruction

33. Denosumab

34. Docetaxel

35. Doxil

36. Doxorubicin

37. Elacestrant

38. Enhertu

39. Epirubicin

40. Eribulin

41. Everolimus

42. Evista

43. Exemestane

44. Exercise

45. Fareston

46. Faslodex

47. Femara

48. Filgrastim

49. Fluorouracil

50. Fulvestrant

51. Gemzar

52. Giredestrant

53. Goserelin

54. Halaven

55. Herceptin

56. Hormonal therapy (oncology)

57. Hyaluronidase

58. Hypnotherapy

59. Ibandronic acid

60. Ibrance

61. Ipatasertib

62. Ixempra

63. Kadcyla

64. Kanjinti

65. Keytruda

66. Kisqali

67. Lapatinib

68. Larotrectinib

69. Lasofoxifene

70. Letrozole

71. Leuprorelin

72. Lumpectomy

73. Lupron

74. Lymphadenectomy

75. Lynparza

76. Margetuximab

77. Massage

78. Mastectomy

79. Medical cannabis

80. Meditation

81. Megace

82. Megestrol

83. Mitoxantrone

84. Navelbine

85. Neratinib

86. Nerlynx

87. Neulasta

88. Neupogen

89. Nivolumab

90. Olaparib

91. Oophorectomy

92. Opdivo

93. Paclitaxel

94. Palbociclib

95. Peer support

96. Pembrolizumab

97. Perjeta

98. Pertuzumab

99. Phesgo

100. Pigray

101. Radiation therapy

102. Raloxifene

103. Ribociclib

104. Rintodestrant

105. Sacituzumab govitecan

106. Talazoparib

107. Talzenna

108. Tamoxifen

109. Targeted therapy

110. Taxol

111. Taxotere

112. Tecentriq

113. Toremifene

114. Trastuzumab

115. Trastuzumab deruxtecan

116. Trodelvy

117. Tucatinib

118. Tukysa

119. Veliparib

120. Verzenio

121. Vinorelbine

122. Xeloda

123. Xgeva

124. Zarxio

125. Zoladex

126. Zoledronic acid

127. Zometa
