# Supplementary material for: Summarizing Online Patient Conversations Using Generative Language Models: Experimental and Comparative Study
Source: JMIR Med Inform. 2025 Apr 14;13:e62909. doi: 10.2196/62909 (PMC12038288; doi:10.2196/62909)
Supplement: Multimedia Appendix 2 [file medinform_v13i1e62909_app2.docx]

## **Multimedia Appendix**

This is a Multimedia Appendix to a full manuscript published in the JMIR Med Inform. For full copyright and citation information see <http://dx.doi.org/10.2196/62909>

**Table 5:** ROUGE Scores and BERTScores obtained for all zero-shot Models without fine-tuning; P values are obtained by comparing to the overall best performing model, i.e., GPT-3.5. Here, B and W indicate the best and worst symptom/treatment number based on each score. The table shows that GPT-3.5 performs best except for ROUGE-2.

| **Zero-shot Models** | **ROUGE-1**  **Mean (SD), B, W** | **ROUGE-2**  **Mean (SD), B, W** | **ROUGE-L**  **Mean (SD), B, W** | **BERTScore**  **Mean (SD), B, W** |
| --- | --- | --- | --- | --- |
| Flan-T5 | 18.91 (±8.14), 105, 107  (*P*<.001) | 3.29 (±4.55), 105, 4  (*P*<.001) | 17.32 (±7.91),  105, 107  (*P*<.001) | 83.34 (±1.85),  3, 82  (*P*<.001) |
| GPT-3 | 32.20 (±11.04),  45, 17  (*P*=.19) | 9.82 (±8.35),  37, 23  (*P*=.63) | 29.10 (±10.94),  45, 59  (*P*=.49) | 87.86 (±3.00) ,  45, 17  (*P*=.35) |
| GPT-3.5 | **34.00** (±11.20),  16, 17 | 10.34 (±8.96),  16, 28 | **30.04** (±11.02),  16, 83 | **88.21** (±3.04),  16, 110 |
| Baseline | 31.67, ±17.24,  6, 67  (*P*=.39) | **13.81**, ±16.5 ,  6, 23  (*P*=.04) | 29.95, ±17.10,  6, 23  (*P*=.94) | 85.47 (± 4.06),  104, 17  (*P*<.001) |

**Table 6:** ROUGE-1/2/L and BERTScore for prompt-based GPT-3.5 models with P values obtained by comparing all prompt-based GPT-3.5 models to the best performing GPT-3.5 DSP three-shot model. Here, B and W indicate the best and worst symptom/treatment number based on each score. The table shows that DSP Three-shot performs best although just with a small margin.

| **Prompt-based GPT-3.5 models** | **ROUGE-1**  **Mean (SD), B, W** | **ROUGE-2**  **Mean (SD), B, W** | **ROUGE-L**  **Mean (SD), B, W** | **BERTScore**  **Mean (SD), B, W** |
| --- | --- | --- | --- | --- |
| Zero-shot | 34.00 (±11.20),  16, 17  (*P*=.55) | 10.34 (±8.96),  16, 28  (*P*=.21) | 30.04 (±11.02),  16, 83  (*P*=.33) | 88.21 (±3.04),  16, 110  (*P*=.98) |
| One-shot | 32.90 (±12. 24),  7, 59  (*P*=.19) | 9.65 (±9.83),  24, 5  (*P*=.08) | 29.01 (±11.61),  7, 59  (*P*=.10) | 88.33 (±3.10),  16, 59  (*P*=.71) |
| Three-shot | 34.87 (±11.93),  89, 67  (*P*=.98) | 11.62 (±10.68),  89, 5  (*P*=.83) | 31.32 (±11.80),  89, 67  (*P*=.93) | **88.44** (±2.91),  15, 17  (*P*=.50) |
| DSP Zero-shot | 34.73 (±11.82),  52, 28  (*P*=.92) | 11.39 (±9.88),  24, 23  (*P*=.68) | 31.02 (±11.35),  16, 83  (*P*=.78) | 88.12 (±2.98),  16, 108  (*P*=.82) |
| DSP One-shot | 34.29 (±12.32),  6, 17  (P=.68) | 10.90 (±10.69),  37, 28  (P=.45) | 30.64 (±12.19),  105, 17  (*P*=.57) | 88.17 ( ±3.04),  104, 108  (*P*=.94) |
| DSP Three-shot | **34.91** (±11.42) **,**  89, 17 | **11.91** (±10.68),  89, 28 | **31.50** (±11.63),  89, 59 | 88.20 (±2.79),  15, 119 |
| CoT Zero-shot | 31.45 (±10.84),  16, 59  (*P*=.01) | 9.23 (±7.72),  16, 28  (*P*=.02) | 28.07 (±10.55),  113, 59  (*P*=.01) | 87.28 (±2.64),  16, 17  (*P*=.009) |

**Table 7:** ROUGE-1/2/L and BERTScore for Flan-T5 Model variants with P values obtained by comparing all Flan-T5 models to the fine-tuned Flan-T5 model. Here, B and W indicate the best and worst symptom/treatment number based on each score. The table shows that the fine-tuned model performs better than all non-fine-tuned models no matter what prompting strategy is used.

| **Flan-T5 Model Variants** | **ROUGE-1**  **Mean (SD), B, W** | **ROUGE-2**  **Mean (SD), B, W** | **ROUGE-L**  **Mean (SD), B, W** | **BERTScore**  **Mean (SD), B, W** |
| --- | --- | --- | --- | --- |
| Zero-shot Flan-T5 | 19.59 (±5.30),  114, 121 (*P*<.001) | 2.82 (±1.52),  115, 121  (*P*<.001) | 18.40 (±5.31),  114,121  (*P*<.001) | 83.69 (±1.57),  125, 122  (*P*<.001) |
| One-shot Flan-T5 | 21.08 (±7.68),  114, 124  (*P*<.001) | 2.88 (±2.63),  116, 117  (*P*=.007) | 18.66 (±7.53),  116, 124  (*P*<.001) | 83.87 (±2.00),  118, 120  (*P*<.001) |
| Three-shot Flan-T5 | 19.96 (±7.82),  117, 124  (*P*<.001) | 3.23 (±2.97),  116, 114  (*P*=0.01) | 17.81 (±6.96),  117, 124  (*P*<.001) | 83.27 (±1.76),  117,121  (*P*<.001) |
| CoT Zero-shot Flan-T5 | 20.00 (±9.26),  127, 119  (*P*<.001) | 2.75 (±2.49),  127, 114  (*P*<.001) | 18.31 (±8.38),  127, 119  (*P*<.001) | 83.36 (±2.52),  118, 119  (*P*<.001) |
| DSP Zero-shot Flan-T5 | 24.84 (±10.29),  117, 119  (*P*=.002) | 6.49 (±7.28),  117, 114  (*P*=.16) | 22.81 (±9.77),  117, 119  (*P*=.003) | 84.05 (±2.98),  117, 119  (*P*<.001) |
| DSP One-shot Flan-T5 | 18.96 (±6.89),  127, 116  (P<.001) | 3.32 (±2.85),  117, 116  (*P*=.09) | 17.83 (±6.90),  127, 116  (P<.001) | 83.30 (±2.10),  117, 116  (P<.001) |
| DSP Three-shot Flan-T5 | 23.60 (±6.25),  116, 124  (*P*=.009) | 4.28 (±4.22),  117, 124  (*P*=.44) | 21.08 (±5.77),  116, 124  (*P*=.02) | 84.38 (±1.86),  118, 125  (P<.001) |
| Fine-tuned Flan-T5 | **30.17** (±6.42),  117, 119 | **7.69** (±4.16),  117, 124 | **27.53** (±5.23),  117, 119 | **86.52** (±1.12),  115, 124 |

Tables 8 and 9 show the average ROUGE-1/2/L and BERTScore for 124 summaries generated by using different prompting techniques on Flan-T5 and GPT-3. In both models, DSP Three-shot prompting shows better performance when compared to other prompting techniques.

**Table 8:** ROUGE-1/2/L and BERTScore obtained for Prompt-based Flan-T5 models in the experiment with P values obtained by comparing all prompt-based Flan-T5 models to the best performing Flan-T5 DSP Three-shot model. Here B and W indicate the best and worst symptom/treatment number based on each score. The table shows that DSP Three-shot performs best.

| **Prompt-based Flan-T5 models** | **ROUGE-1**  **Mean (SD),B,W** | **ROUGE-2**  **Mean (SD), B,W** | **ROUGE-L**  **Mean (SD),B, W** | **BERTScore**  **Mean (SD),B, W** |
| --- | --- | --- | --- | --- |
| Zero-shot | 18.91 (±8.14),  105, 107  (*P*<.001) | 3.29 (±4.55),  105, 4  (*P*<.001) | 17.32 (±7.91),  105, 107  (*P*<.001) | 83.34 (±1.85),  3, 82  (*P*= .63) |
| One-shot | 19.83 (±8.76),  3, 45  (*P*=.005) | 3.84 (±5.06),  3, 21  (*P*=.008) | 18.29 (±8.26),  3, 45  (*P*=.005) | 83.28 (±2.07),  37, 112  (*P*= .52) |
| Three-shot | 20.50 (±8.86),  37, 59  (*P*=.02) | 3.95 (±5.33),  37, 11  (*P*=.01) | 18.44 (±8.07),  37, 59  (*P*=.007) | 83.40 (±1.96),  29, 76  (*P*=.80) |
| DSP Zero-shot | 22.54 (±8.77),  117, 67  (*P*=.60) | 5.35 (±5.99),  117, 21  (*P*=.42) | 20.57 (±8.40),  117, 67  (*P*=.41) | **83.54** (±2.27),  117, 124  (*P*=.79) |
| DSP One-shot | 21.52 (±8.37),  96, 116  (*P*=.16) | 5.06 (±5.56),  96, 10  (*P*=.25) | 19.85 (±8.21),  96, 116  (*P*=.14) | 83.42 (±2.17),  3, 24  (*P*=.89) |
| DSP Three-shot | **23.14** (±9.69),  29, 59 | **6.05** (±7.73),  29, 20 | **21.50** (±9.57),  29, 59 | 83.46 (±2.28),  29, 52 |
| CoT Zero-shot | 18.34 (±8.84),  105, 119  (*P*<.001) | 2.95 (±4.16),  105, 5  (*P*<.001) | 16.76 (±8.32),  105, 119  (*P*<.001) | 82.90 (±2.08),  118, 20  (*P*=.04) |

**Table 9:** ROUGE-1/2/L and BERTScore obtained for Prompt-based GPT-3 models in the experiment with P values obtained by comparing all prompt-based GPT-3 models to the best performing GPT-3 DSP Three-shot model. Here B and W indicate the best and worst symptom/treatment number based on each score. The table shows that DSP Three-shot performs best.

| **Prompt-based GPT-3 models** | **ROUGE-1**  **Mean (SD),B, W** | **ROUGE-2**  **Mean (SD),B, W** | **ROUGE-L**  **Mean (SD),B, W** | **BERTScore**  **Mean (SD),B, W** |
| --- | --- | --- | --- | --- |
| Zero-shot | 32.20 (±11.04),  45, 17  (*P*=.14) | 9.82 (±8.35),  37, 23  (*P*=.61) | 29.10 (±10.94),  45, 59  (*P*=.40) | 87.86 (±3.00),  45, 17  (*P*=.67) |
| One-shot | 33.00 (±11.11),  89, 83  (*P*=.38) | 9.50 (±7.88),  24, 28  (*P*=.39) | 28.93 (±10.25),  89, 83  (*P*=.30) | **88.31** (±2.90),  16, 108  (*P*=.39) |
| Three-shot | 33.91 (±9.37),  6, 17  (*P*=.83) | 9.19 (±6.51),  37, 23  (*P*=.20) | 29.47 (±8.65) ,  68, 59  (*P*=.51) | 88.06 (±2.71),  16, 18  (*P*=.86) |
| DSP Zero-shot | 32.43(±10.95),  52, 67  (*P*=.21) | 9.52 (±8.47),  104, 4  (*P*=.44) | 28.97 (±11.00),  52, 67  (*P*=.37) | 87.54 (±2.79),  16, 23  (*P*=.19) |
| DSP One-shot | 33.03 (±11.34),  37, 67  (*P*=.42) | 9.68 (±8.67),  37, 23  (*P*=.53) | 29.65 (±11.12),  37, 83  (*P*=.68) | 88.00 (±2.91),  16, 19  (*P*=.99) |
| DSP Three-shot | **34.20**(±10.51),  37, 17 | **10.34** (±8.09),  37, 5 | **30.23**(±10.01),  37, 17 | 88.00 (±2.64),  16, 17 |
| CoT Zero-shot | 31.74 (±11.72),  105, 67  (*P*=.08) | 10.27 (±9.89),  105, 5  (*P*=.94) | 28.78 (±11.55),  105, 67  (*P*=.30) | 87.45 (±2.98),  15, 60  (*P*=.12) |

The prompt templates for each prompting technique used in this research are given below.

In the given prompt templates {text}denotes the patient comment regarding a symptom or treatment drawn from the example set, {hint}denotes hints which are a combination of keywords that are extracted by KeyBERT with KeyphraseVectorizers and frequent terms that occur in each patient comment, {A}denotes the generative language model generated answer for each question and {summary}denotes the reference summary of the corresponding patient comment.

**Zero-shot prompt template:**

Write a short summary for this patient comment: {text}

Summary:

**One-shot prompt template:**

Given a patient comment, write a short summary for the patient comment.

Write a short summary for this patient comment: {text}

Summary: {summary}

Write a short summary for this patient comment: {text}

Summary:

Figure 4: One-shot prompt template used in the experiments.

**Three-shot prompt template:**

Given a patient comment, write a short summary for the patient comment.

Write a short summary for this patient comment: {text}

Summary: {summary}

Write a short summary for this patient comment: {text}

Summary: {summary}

Write a short summary for this patient comment: {text}

Summary: {summary}

Write a short summary for this patient comment: {text}

Summary:

**Directional Stimulus prompt - Zero-shot template:**

Write a short summary based on the provided hint for this patient comment: {text}

Hint: {hint}

Summary:

**Directional Stimulus prompt - One-shot template:**

Given a patient comment and hint, write a short summary for the patient comment based on the hint.

Write a short summary based on the provided hint for this patient comment: {text}

Hint:{hint}

Summary: {summary}

Write a short summary based on the provided hint for this patient comment: {text}

Hint: {hint}

Summary:

**Directional Stimulus prompt - Three-shot template:**

Given a patient comment and hint, write a short summary for the patient comment based on the hint.

Write a short summary based on the provided hint for this patient comment: {text}

Hint:{hint}

Summary: {summary}

Write a short summary based on the provided hint for this patient comment: {text}

Hint:{hint}

Summary: {summary}

Write a short summary based on the provided hint for this patient comment: {text}

Hint:{hint}

Summary: {summary}

Write a short summary based on the provided hint for this patient comment: {text}

Hint: {hint}

Summary:

**Chain-of-Thought Zero-shot prompt template - Flan-T5:**

What is the main health or medical related topic discussed in this patient comment?: {A}

What are the important informations mentioned in this patient comment?: {A}

What are the symptoms and diseases mentioned in this patient comment?: {A}

What are the recovery methods or treatments mentioned in this patient comment?: {A}

Let's integrate the above information and summarize this patient comment: {text}

Summary:

**Chain-of-Thought Zero-shot prompt template - GPT-3 & GPT-3.5:**

1. The main health or medical related topic {A}.

2. The important information mentioned {A}.

3. The diseases mentioned {A}.

4. The recovery methods or treatments mentioned {A}.

Let's integrate the above information and summarize the patient comment: {text}

Summary:
